# The Fear of COVID-19 Scale: Development and Initial Validation

**DOI:** 10.1007/s11469-020-00270-8

**Published:** 2020-03-27

**Authors:** Daniel Kwasi Ahorsu, Chung-Ying Lin, Vida Imani, Mohsen Saffari, Mark D. Griffiths, Amir H. Pakpour

**Affiliations:** 1 Department of Rehabilitation Sciences, The Hong Kong Polytechnic University, Hung Hom, Hong Kong; 2 Pediatric Health Research Center, Tabriz University of Medical Sciences, Tabriz, Iran; 3 Health Research Center, Life Style Institute, Baqiyatallah University of Medical Sciences, Tehran, Iran; 4 International Gaming Research Unit, Psychology Department, Nottingham Trent University, 50 Shakespeare Street, Nottingham, NG1 4FQ UK; 5 Social Determinants of Health Research Center, Research Institute for Prevention of Non-Communicable Diseases, Qazvin University of Medical Sciences, Shahid Bahonar Blvd., Qazvin, 3419759811 Iran; 6 Department of Nursing, School of Health and Welfare, Jönköping University, Jönköping, Sweden

**Keywords:** COVID-19, Fear, Iran, Psychometrics, Fear of COVID-19 Scale

## Abstract

**Background:**

The emergence of the COVID-19 and its consequences has led to fears, worries, and anxiety among individuals worldwide. The present study developed the Fear of COVID-19 Scale (FCV-19S) to complement the clinical efforts in preventing the spread and treating of COVID-19 cases.

**Methods:**

The sample comprised 717 Iranian participants. The items of the FCV-19S were constructed based on extensive review of existing scales on fears, expert evaluations, and participant interviews. Several psychometric tests were conducted to ascertain its reliability and validity properties.

**Results:**

After panel review and corrected item-total correlation testing, seven items with acceptable corrected item-total correlation (0.47 to 0.56) were retained and further confirmed by significant and strong factor loadings (0.66 to 0.74). Also, other properties evaluated using both classical test theory and Rasch model were satisfactory on the seven-item scale. More specifically, reliability values such as internal consistency (*α* = .82) and test–retest reliability (ICC = .72) were acceptable. Concurrent validity was supported by the Hospital Anxiety and Depression Scale (with depression, *r* = 0.425 and anxiety, *r* = 0.511) and the Perceived Vulnerability to Disease Scale (with perceived infectability, *r* = 0.483 and germ aversion, r = 0.459).

**Conclusion:**

The Fear of COVID-19 Scale, a seven-item scale, has robust psychometric properties. It is reliable and valid in assessing fear of COVID-19 among the general population and will also be useful in allaying COVID-19 fears among individuals.

One of the emergent global challenges in managing infectious diseases is dealing with the novel coronavirus 2019 (COVID-19). The most common symptoms within 2–14 days include fever, fatigue, dry cough, myalgia, and dyspnea (Wang et al. 2020). As of March 1, 2020, the mortality rate was 3.6% in China and 1.5% outside China (Baud et al. 2020), and as of March 14, 2020, 135 countries/territories had confirmed cases (World Health Organization 2020). With the extremely high infection rate and relatively high mortality, individuals naturally began worrying about the COVID-19. Indeed, fear of contacting individuals who are possibly infected by COVID-19 has been reported (Lin 2020). Unfortunately, fear may amplify the damage of the disease itself. The emergence of the COVID-19 (Guan et al. 2020; Huang et al. 2020) and its pandemic nature has exacerbated fears worldwide leading to stigma in some cases (Centers for Disease Control and Prevention 2020a, b; Lin 2020). One characteristic nature of infectious disease compared with other conditions is fear. Fear is directly associated with its transmission rate and medium (rapidly and invisibly) as well as its morbidity and mortality. This further leads to other psychosocial challenges including stigmatization, discrimination, and loss (Pappas et al. 2009). With the high levels of fear, individuals may not think clearly and rationally when reacting to COVID-19.

However, current treatment on COVID-19 worldwide has mainly focused on infection control, effective vaccine, and treatment cure rate (Dong et al. 2020; Wang et al. 2020). The psychosocial aspect has yet to be thoroughly considered. Nevertheless, as countries worldwide have to work on reducing the transmission rate of COVID-19, they should also work on individual fears to achieve the holistic goal of having a society free of COVID-19. One of the reasons that current treatment on COVID-19 pays little attention to the fear of COVID-19 is the lack of an appropriate psychometric instrument. Therefore, developing a brief and valid instrument to capture an individual’s fear of COVID-19 is both timely and important. With the information on how an individual fears COVID-19, healthcare providers can further design appropriate programs to take care of the fear. Therefore, this study developed and validated a scale assessing the fear of coronavirus—the Fear of COVID-19 Scale (FCV-19S) (see “Appendix” section)—using two types of psychometric testing: classical test theory (CTT) analysis and Rasch analysis. Consequently, the FCV-19S will be useful in providing valuable information on fear of COVID-19 so as to facilitate public health initiatives on allaying public’s fears.

## Methods

### Development of the Scale

Several steps were taken to develop the Fear of COVID-19 Scale (McCoach et al. 2013). First, an extensive literature review was conducted to assess all general scales on fear. Thirty measures on fear were identified that assess fear on different populations and diseases (available on request from the corresponding authors). Relevant and possible items were pooled by two researchers (i.e., the third and last authors). After removing those items with similar content or expressions, 28 items were retained for further evaluation. Second, an expert panel (comprising a psychologist, virologist, health psychologist, psychiatrist, general physician, and nurse) evaluated the 28 items, and 11 items were deleted based on the suggestion from the expert panel. Third, the retained 17 items were sent out to a different expert panel (comprising a health education specialist, pulmonologist, social psychologist, and sociologist in Iran) to review. Seven items were further omitted based on the comments from the second expert panel. Finally, the 10-item scale was piloted on 46 individuals (26 males and 20 females, mean age 39.63 years, number of years in education = 9.38 years) to obtain initial assessment of the scale. A four-point Likert scale was used to test whether the individuals understand the item descriptions. The results showed that all respondents fully understood the item descriptions (mean 3.81, SD = 1.04). Additionally, an individual telephone-based cognitive interview was implemented on the same pilot participants to explore their thoughts about each scale item and their responses. No further changes were made because the pilot participants indicated no changes were needed.

### Participants and Procedure

The target population was the general Iranian population. Inclusion criteria were being (i) an Iranian, (ii) aged 18 years or older, and (iii) being able to understand spoken Persian or Farsi. Participants were recruited from online advertisements, e-mail campaigns, blogs, social media, and SMS campaigns. All procedures conducted were approved by the Ethics Committee of Qazvin University of Medical Sciences (IR.QUMS.REC.1398.375). Informed consent was obtained electronically before data were collected from the participants.

## Measures

### Demographic Information

A background information sheet consisting of age, educational year, gender, and whether they were a current smoker was used to obtain demographic information about the participants.

### Hospital Anxiety and Depression Scale

The Persian version of Hospital Anxiety and Depression Scale (HADS) was used to assess the anxiety and depression levels of participants. The HADS is a 14-item scale in which items are answered in a four-point response format with a total score ranging from 0 to 21 for each of the subscales (anxiety and depression with seven items each). Example items include *“I have lost interest in my appearance”* (depression) and *“Worrying thoughts go through my mind” *(anxiety). A higher score is indicative of severe anxiety or depression. The scale has acceptable reliability (Cronbach’s *α* of 0.78 and 0.86 for the HADS anxiety and depression respectively) and a satisfactory validity (Montazeri et al. 2003).

### Perceived Vulnerability to Disease Scale

The Persian version of the 15-item Perceived Vulnerability to Disease Scale (PVDS) was used to assess participant’s perceived vulnerability to infectious disease specifically perceived infectability (seven-item subscale) and germ aversion (eight-item subscale). Participants respond to each item on a seven-point scale (from “strongly disagree” to “strongly agree”) with approximately half of the items reverse-scored (Ahmadzadeh et al. 2013; Duncan et al. 2009). Example items include *“In general, I am very susceptible to colds, flu and other infectious diseases” *(perceived infectability) and *“I prefer to wash my hands pretty soon after shaking someone’s hand” *(germ aversion). A higher score indicates a severe form of perceived infectability, germ aversion, or perceived vulnerability to disease (as a whole). It has a Cronbach’s *α* of 0.70, 0.72, and 0.70 for perceived infectability, germ aversion, and total PVDS score respectively (Ahmadzadeh et al. 2013).

### Data Analysis

Descriptive statistics were used to understand participants’ characteristics. Analyses on psychometric properties included CTT analysis and Rasch model analysis. CTT analysis included internal consistency, test-retest reliability, corrected item-total correlation, average variance extracted (AVE), composite reliability, standard error of measurement, concurrent validity, and exploratory factor analysis (EFA). Rasch model analysis included infit and outfit mean square (MnSq) for each item, item and person separation reliability, and item and person separation index. Also, differential item functioning (DIF) based on Rasch analysis was used to test the measurement invariance across gender and age (Wu et al. 2017). All the descriptive and CTT analyses were conducted using IBM SPSS 23.0 (IBM Corp., Armonk, NY). Also, the Rasch model analyses were conducted using WINSTEPS 3.75.0.

## Results

The mean age of the participants (*n* = 717) was 31.25 years (SD ± 12.68), and the participants had, on average, 8.9 years of education (SD ± 4.1). More than half of the participants were males (*n* = 416; 58%), and nearly one-fifth of the participants were current smokers (*n* = 139; 19.4%). According to the corrected inter-item correlation, three items with low corrected item-total correlations were deleted (i.e., Item 3, − 0.06; Item 4, − 0.03; Item 5 − 0.05). The remaining seven items in the scale all had acceptable corrected item-total correlation (0.47 to 0.56) and were used for following psychometric testing (Table 1).

**Table 1:** Descriptive statistics and item-total correlation of the Fear of COVID-19 Scale (*n* = 717)

Item number	Mean (SD)	Corrected item-total correlation	Skewness	Kurtosis	Missing data (%)	Floor effect (%)	Ceiling effect (%)	Item exclusion or retention
Item 1. I am most afraid of coronavirus-19.	3.48 (1.14)	0.47	− 0.34	− 0.61	6.69	5.7	22.2	Retained
Item 2. It makes me uncomfortable to think about coronavirus-19.	4.01 (0.84)	0.56	− 0.58	− 0.12	3.21	4.9	30.1	Retained
Item 3. I worry a lot about coronavirus-19.	3.43 (1.23)	− 0.06	− 2.41	− 1.91	5.16	7.3	21.3	Excluded
Item 4. Coronavirus-19 is almost always terminal	2.62 (1.16)	− 0.03	0.24	− 2.12	9.34	18.4	3.8	Excluded
Item 5. Coronavirus-19 is an unpredictable disease	3.91 (0.96)	− 0.05	− 1.66	− 1.07	10.32	7.1	30.5	Excluded
Item 6. My hands become clammy when I think about coronavirus-19	3.76 (0.88)	0.51	− 0.42	0.12	3.21	0.7	47.6	Retained
Item 7. I am afraid of losing my life because of coronavirus-19	4.24 (0.90)	0.54	−1.18	0.98	3.07	0.4	34.3	Retained
Item 8.When watching news and stories about coronavirus-19 on social media, I become nervous or anxious.	3.53 (1.07)	0.51	− 0.36	− 0.47	2.51	1.4	20.5	Retained
Item 9. I cannot sleep because I’m worrying about getting coronavirus-19	4.11 (0.81)	0.48	− 0.79	0.56	5.43	3.9	20.8	Retained
Item 10. My heart races or palpitates when I think about getting coronavirus-19	4.26 (0.75)	0.53	− 0.91	0.78	5.86	2.5	42.1	Retained

All factor loadings from the retained seven items were significant and strong (0.66 to 0.74). Their properties tested using Rasch analysis were satisfactory: infit MnSq values were between 0.80 and 1.26; outfit MnSq values were between 0.84 and 1.25. No substantial DIF was found across gender and age (Table 2). Table 3 demonstrates that the seven-item scale had robust psychometric properties in the scale level. From CTT analysis, the internal consistency was good (*α* = .82), composite reliability (0.88) and AVE (0.51) were acceptable, and standard error of measurement was low (1.89). From Rasch analysis, item separation reliability (0.99), item separation index (11.45), person separation reliability (0.77), and the person separation index (2.82) were all satisfactory (Table 3).

**Table 2:** Psychometric properties of the Fear of COVID-19 Scale at the item level (*n* = 717)

Item number	Factor loading*	*h*^2^	Infit MnSq	Outfit MnSq	Difficulty	DIF contrast across gender^cd^	DIF contrast across age^ce^
Item 1. I am most afraid of coronavirus-19.	0.66	0.38	1.26	1.25	0.98	− 0.10	− 0.05
Item 2. It makes me uncomfortable to think about coronavirus-19.	0.74	0.55	0.80	0.84	− 0.17	− 0.33	− 0.22
Item 6. My hands become clammy when I think about coronavirus-19.	0.71	0.51	0.81	0.85	0.39	− 0.29	0.25
Item 7. I am afraid of losing my life because of coronavirus-19.	0.74	0.55	1.11	1.0	−0.77	0.29	0.21
Item 8. When watching news and stories about coronavirus-19 on social media, I become nervous or anxious.	0.71	0.50	1.01	1.0	0.85	0.48	0.30
Item 9. I cannot sleep because I’m worrying about getting coronavirus-19.	0.71	0.50	0.90	0.94	− 0.43	− 0.24	− 0.31
Item 10. My heart races or palpitates when I think about getting coronavirus-19.	0.73	0.54	0.81	0.91	− 0.83	0.21	− 0.23

*MnSq* mean square error, *DIF* differential item functioning

*Extraction method: Oblimin rotation with Kaiser normalization. *h*2 = communalities

^c^DIF contrast > 0.5 indicates substantial DIF

^d^DIF contrast across gender = difficulty for males-difficulty for females

^e^DIF contrast across age groups = Difficulty for younger (i.e., ≤ 31.25 years) - Difficulty for older (i.e., > 31.25 years) patients

**Table 3:** Psychometric properties of the Fear of COVID-19 Scale at the scale level (*n* = 717)

Psychometric testing	Value	Suggested cutoff
Composite reliability	0.88	> 0.7
Average variance extracted	0.51	> 0.5
Internal consistency (Cronbach’s *α*)	0.82	> 0.7
Standard error of measurement	1.89	The smaller the better
Item separation reliability from Rasch	0.99	> 0.7
Item separation index from Rasch	11.45	> 2
Person separation reliability from Rasch	0.77	> 0.7
Person separation index from Rasch	2.82	> 2

Concurrent validity was supported by the HADS and PVDS as indicated by the significant correlations (both *p* < 0.001). There were significant positive correlations between fear of COVID-19 and (i) depression (*r* = 0.425, *p* < 0.001), (ii) anxiety (*r* = 0.511, *p* < 0.001), (iii) perceived infectability (*r* = 0.483, *p* < 0.001), and (iv) germ aversion (*r* = 0.459, *p* < 0.001).

## Discussion

The present study presented the development of a new scale, the Fear of COVID-19 Scale (FC-19S). Findings demonstrated that the FCV-19S has a stable unidimensional structure with robust psychometric properties. Initial psychometric results indicated that the FCV-19S had good properties from different types of testing (i.e., CTT and Rasch analysis). Moreover, the overall score of the summed-up items scores can indicate the severity of the fear of COVID-19. More specifically, the higher the score on the FCV-19S, the higher the scores on the HADS and the PVDS were. Therefore, higher overall scores on the FCV-19S indicate more severe fear of COVID-19. Also, gender and age appeared not to affect the response pattern of the fear on the FCV-19S. Therefore, it can be concluded that the FCV-19S can be relied upon to assess and deal with the psychological issues emanating from COVID-19 among males and females as well as individuals of all ages.

Other studies have shown that negative effects of psychological reactions such as hypochondriasis and anxiety affect individuals’ health and well being during times of infectious epidemic crisis (Duncan et al. 2009; Pappas et al. 2009; Ropeik 2004). The FCV-19S could also help allay the fears of the public on COVID-19 and subsequently help in reducing the stigma, anxiety, and stress attached to it (Centers for Disease Control and Prevention 2020a, b). The concurrent validity, ascertained using the HADS and PVDS, indicates that the FCV-19S is a valid tool for assessing fear of COVID-19. Also, the FCV-19S significantly correlated with depression, anxiety (on the HADS), perceived infectability, and germ aversion (on the PVDS) which suggests that individuals with severe fear of COVID-19 may have these comorbid disorders.

The present study has some limitations. First, the studied participants were from the general Iranian population and no formal diagnoses on mood disorders were obtained (e.g., anxiety). Therefore, the sensitivity and specificity of the scale cannot be examined. Second, although fear is subjective and has shortcomings when trying to assess it objectively, the nature of the self-report cannot exclude the possibility that respondents provided responses affected by social desirability factors. Third, convenience sampling weakens the generalizability of the findings in the present study. Finally, the single-factor structure of the scale was based on EFA and Rasch analysis; consequently, further verification, such as using confirmatory factor analysis, on its factor structure is needed.

In conclusion, this study demonstrated that the Fear of COVID-19 Scale is a seven-item unidimensional scale with robust psychometric properties. Moreover, total scores on the FCV-19S are comparable across both genders and all ages which suggest that it is a good psychometric instrument to be used in assessing and allaying fears of COVID-19 among individuals.

## Appendix

Fear of Coronavirus-19 Scale

1. I am most afraid of coronavirus-19.
2. It makes me uncomfortable to think about coronavirus-19.
3. My hands become clammy when I think about coronavirus-19.
4. I am afraid of losing my life because of coronavirus-19.
5. When watching news and stories about coronavirus-19 on social media, I become nervous or anxious.
6. I cannot sleep because I’m worrying about getting coronavirus-19.
7. My heart races or palpitates when I think about getting coronavirus-19.

The participants indicate their level of agreement with the statements using a five-item Likert-type scale. Answers included “strongly disagree,” “disagree,” “neither agree nor disagree,” “agree,” and “strongly agree”. The minimum score possible for each question is 1, and the maximum is 5. A total score is calculated by adding up each item score (ranging from 7 to 35). The higher the score, the greater the fear of cororonavirus-19.
